# Accuracy of Magnetometer-Guided Sentinel Lymphadenectomy after Intraprostatic Injection of Superparamagnetic Iron Oxide Nanoparticles in Prostate Cancer: The SentiMag Pro II Study

**DOI:** 10.3390/cancers12010032

**Published:** 2019-12-20

**Authors:** Alexander Winter, Svenja Engels, Philipp Goos, Marie-Christin Süykers, Stefan Gudenkauf, Rolf-Peter Henke, Friedhelm Wawroschek

**Affiliations:** 1 University Hospital for Urology, Klinikum Oldenburg, School of Medicine and Health Sciences, Carl von Ossietzky University Oldenburg, D-26111 Oldenburg, Germany; engels.svenja@klinikum-oldenburg.de (S.E.); philipp.goos@web.de (P.G.); marie_chr.sueykers@yahoo.de (M.-C.S.); wawroschek.friedhelm@klinikum-oldenburg.de (F.W.); 2 Departments of Business Information Systems, University of Applied Sciences and Arts Hannover, D-30459 Hannover, Germany; stefan.gudenkauf@hs-hannover.de; 3 Institute of Pathology Oldenburg, D-26122 Oldenburg, Germany; r.p.henke@pathologie-oldenburg.de

**Keywords:** lymphadenectomy, magnetometer, prostate cancer, sentinel lymph node dissection, SPION, superparamagnetic iron oxide nanoparticles

## Abstract

Radioisotope-guided sentinel lymph node dissection (sLND) has shown high diagnostic reliability in prostate (PCa) and other cancers. To overcome the limitations of the radioactive tracers, magnetometer-guided sLND using superparamagnetic iron oxide nanoparticles (SPIONs) has been successfully used in PCa. This prospective study (SentiMag Pro II, DRKS00007671) determined the diagnostic accuracy of magnetometer-guided sLND in intermediate- and high-risk PCa. Fifty intermediate- or high-risk PCa patients (prostate-specific antigen (PSA) ≥ 10 ng/mL and/or Gleason score ≥ 7; median PSA 10.8 ng/mL, IQR 7.4–19.2 ng/mL) were enrolled. After the intraprostatic SPIONs injection a day earlier, patients underwent magnetometer-guided sLND and extended lymph node dissection (eLND, followed by radical prostatectomy. SLNs were detected in in vivo and in ex vivo samples. Diagnostic accuracy of sLND was assessed using eLND as the reference. SLNs were detected in all patients (detection rate 100%), with 447 sentinel lymph nodes SLNs (median 9, IQR 6–12) being identified and 966 LNs (median 18, IQR 15–23) being removed. Thirty-six percent (18/50) of patients had LN metastases (median 2, IQR 1–3). Magnetometer-guided sLND had 100% sensitivity, 97.0% specificity, 94.4% positive predictive value, 100% negative predictive value, 0.0% false negative rate, and 3.0% additional diagnostic value (LN metastases only in SLNs outside the eLND template). In vivo, one positive SLN/LN-positive patient was missed, resulting in a sensitivity of 94.4%. In conclusion, this new magnetic sentinel procedure has high accuracy for nodal staging in intermediate- and high-risk PCa. The reliability of intraoperative SLN detection using this magnetometer system requires verification in further multicentric studies.

## 1. Introduction

Histopathological examination or pelvic lymph node (LN) dissection (LND) is still the gold standard for LN staging in clinically localized prostate cancer (PCa). The number of removed LNs or the extent of LND directly influence the rate of detected LN-positive patients [1]. On the other hand, complications arise along with the number of LNs removed [2].

Because of relevant importance for further therapy and the complication rate of extended LND (eLND), as well as the low detection rate of limited LND methods, the concept of targeted radioisotope-guided sentinel LN (SLN) identification used in other tumor entities was implemented in PCa [3]. Conventionally, marking of SLNs with technetium-99m (^99m^Tc) nanocolloid and a gamma probe for intraoperative SLN detection are used for the established radioisotope-guided SLN identification in PCa patients. The diagnostic accuracy of this radioactive sentinel LN dissection (sLND) approach was determined in a systematic literature review. Twenty-one studies including 2509 patients were analyzed [4]. The findings demonstrated that the diagnostic reliability of eLND and sLND were almost comparable. In sentinel cohorts, targeted sLND detected a higher rate of LN-positive patients than were expected from established nomograms [5,6,7].

Because of the ionizing radiation emitted by the radioactive tracer material, the benefits of the established SLN procedure are associated with certain limitations. The dependence on radioisotopes or nuclear medicine facilities imposes restrictions on patient planning and hospital logistics. In principle, the application of this procedure is thus limited to small parts of the developed world. Moreover, surgical staff and patients are exposed to ionizing radiation.

Superparamagnetic iron oxide nanoparticles (SPIONs) have received much attention in bioscientific research since the first report in the 1980s and have increasingly been used clinically in recent years. Biocompatible SPIONs with a suitable surface architecture have triggered research efforts for both cellular imaging and drug-delivery applications. One of the main features of SPIONs is the ability to show magnetization only in an applied magnetic field. SPIONs have the ability to form stable colloidal suspensions, which is crucial for in vivo biomedical applications [8]. SPIONs enhance both T1 and T2/T2* relaxation. In consequence, the uptake of SPION contrast agents results in a drop of signal intensity (‘negative contrast’) on T2* (susceptibility)-weighted magnetic resonance imaging (MRI) sequences [9]. In conjunction with a particle size optimized for filtration and retention by SLNs, these characteristics enable preoperative visualization of SLNs using MRI before intraoperative detection by handheld magnetometers [10,11]. The natural dark brown color can help to further identify SLNs intraoperatively and eliminates the need for separate (carbon or blue) dye injections in sentinel-guided surgery.

Based on these advantageous properties, SPIONs have been successfully applied for marking and intraoperative detection of SLNs in breast cancer to overcome the drawbacks of the radioisotope-based sentinel procedure [12]. In a pilot study, we presented the first results on the intraoperative detection of SLNs in PCa patients using a handheld magnetometer system after intraprostatic injection of SPIONs and demonstrated the feasibility and safety of this magnetic SLN identification procedure in PCa [13].

In view of these findings, we hypothesized that magnetometer-guided sLND based on intraoperative identification of SPION-marked SLNs would have high reliability in the identification of LN-positive PCa patients, being comparable with the radioisotope-guided sentinel procedure.

To assess the diagnostic accuracy of magnetometer-guided sLND in PCa, this prospective single-center study analyzed intermediate- and high-risk PCa patients who underwent magnetometer-guided sLND and eLND, followed by radical retropubic prostatectomy. The diagnostic accuracy of magnetometer-guided sLND was determined using eLND as the reference standard.

## 2. Results

As planned, the study included 50 intermediate- or high-risk PCa patients who underwent radical retropubic prostatectomy with magnetometer-guided sLND after intraprostatic injection of SPIONs, with eLND being performed as the reference standard. Table 1 summarizes the patient characteristics.

In all, 966 LNs (median 18 per patient, interquartile range (IQR) 15–23) were removed. At least one SLN was successfully detected by magnetometer-guided sLND in all patients (50/50), resulting in a detection rate of 100%. According to the ex vivo measurements of magnetic LN activity, a total of 447 SLNs were identified. The median number of detected SLNs was 9 (IQR 6–12).

**Figure 1:**
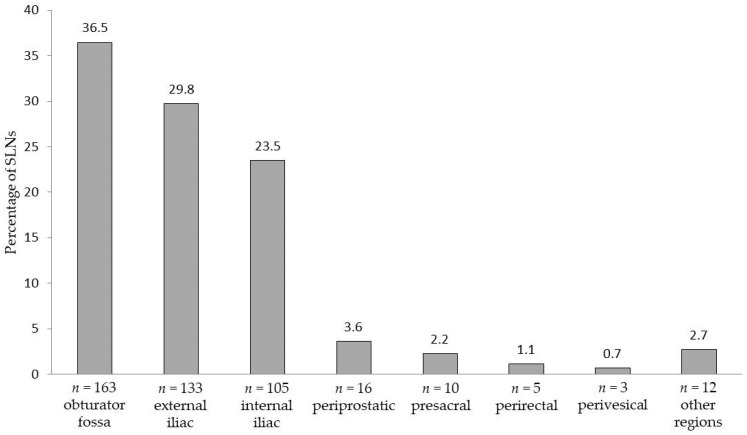
Areas and anatomical distribution of the 447 prostate sentinel lymph nodes from the 50 intermediate- or high-risk patients based on magnetometer-guided detection after intraprostatic injection of superparamagnetic iron oxide nanoparticles.

SLNs were also localized outside the established eLND template (e.g., the periprostatic region: 3.6%; presacral region: 2.2%). Figure 1 shows the detailed distributions of all SLNs per anatomical region.

**Figure 2:**
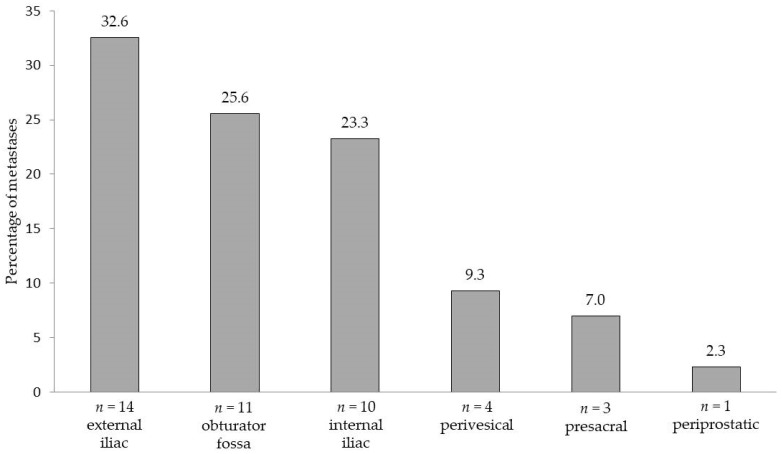
Areas and anatomical distribution of lymph node metastases (*n* = 43) detected by extended pelvic lymph node dissection and/or magnetometer-guided sentinel lymphadenectomy after intraprostatic injection of superparamagnetic iron oxide nanoparticles in 18 lymph node-positive patients with intermediate- or high-risk prostate cancer.

LN metastases were found in 36% (18/50) of patients. In total, 43 LNs were metastasis-positive, with the median number of positive nodes (when present) being 2 (IQR 1–3). Taking eLND as the reference standard, the sensitivity of the magnetic SLN procedure was 100%, i.e., all patients with LN metastases were correctly detected as LN-positive. The magnetometer-guided sLND results had a specificity of 97.0%, positive predictive value (PPV) of 94.4%, and negative predictive value (NPV) of 100%, resulting in a false negative rate of 0.0%. sLND was shown to be of additional diagnostic value in one of the 18 LN-positive patients. In this case, sLND resulted in the detection of one LN metastasis outside the eLND template (presacral), while eLND did not reveal any metastases (false positive rate 3%). Figure 2 shows the distribution of all detected LN metastases per anatomical region.

The percentage of LN-positive patients with metastases only in SLNs was 77.8% (*n* = 14).

Intraoperative measurement of magnetic activity or detection of SLNs using the handheld magnetometer missed one LN-positive patient, in whom one positive SLN was not detected, resulting in a sensitivity of 94.4% (17/18).

## 3. Discussion

After the successful application of the magnetometer-guided sentinel approach in breast cancer, the feasibility and safety of intraoperative detection of SPION-marked SLNs using a handheld magnetometer following intraprostatic SPIONs injection was demonstrated in PCa [13]. Currently, the use of this magnetic sentinel procedure is also being investigated in other tumor entities, for example, initial positive results have recently been shown for penile cancer [14].

On the basis of results comparable to the radioactive marking of SLNs in breast cancer and the promising first results presented in our PCa pilot study (SentiMag Pro I) [12,13,15], we hypothesized that magnetometer-guided sLND would also have high reliability in the identification of SLNs or LN-positive PCa patients, being comparable to the radioisotope-guided sentinel approach.

In the results presented for the SentiMag Pro II trial, which included PCa patients with an intermediate- or high-risk for the presence of lymphatic metastasis, SLNs were identified in all patients, resulting in a detection rate of 100%. This is better than in our pilot study, that included PCa patients with the same risk factors, where the magnetic technique successfully identified SLNs in only 89.5% of cases [13]. For radioisotope-guided sLND, a detection rate of 98.0% was reported in a study including over 2000 low-, intermediate-, and high-risk PCa patients [16]. One meta-analysis demonstrated a pooled detection rate of 93.8% for the radio-guided sentinel approach [17]. A systematic literature review considering 21 studies recruiting 2509 patients, found a median cumulative percentage detection rate of 95.9% (IQR 89.4–98.5%) [4]. However, in the SentiMag Pro II study, we adjusted the exclusion criteria according to some of the fundamental limitations of sLND already described by us and others (e.g., previous hormonal treatment or prostate surgery), which may have improved our detection rate [13].

In the ex vivo analysis using the handheld magnetometer to identify SLNs, all LN-positive patients were correctly detected in the SentiMag Pro II study. However, one metastatic SLN was not detected intracorporeally using the SentiMag probe, resulting in the missing of one LN-positive patient and a sensitivity of 94.4%. This patient had a high-risk tumor with large volume (Gleason score 4 + 5, pT3b (Union for International Cancer Control (UICC) tumor–node–metastasis (TNM) classification of malignant tumors, 7th Edition), PSA 44 ng/mL), which may have obstructed the outflow of the tracer. In vivo, only one SLN showing weakly magnetic activity (11 counts) could be identified using the SentiMag probe. In ex vivo measurement, magnetic activity was detected in five of the 35 LNs removed, which, like the metastasis-positive SLN overlooked intraoperatively (50 counts), had a comparatively low activity. The positive SLN, which was not detected intraoperatively, was located in the deep presacral area, so that the limitations of the intracorporeal detection of SPION-marked SLNs described in the next but one paragraph may have become noticeable, taking into account the weak activity.

In the systematic literature review mentioned above, the median cumulative percentage results for sLND showed a sensitivity of 95.2% (81.8–100%) and false negative rate of 4.8% (0–18.2%), taking into account in vivo SLN identification [4]. Accordingly, the SentiMag Pro II results can be considered comparable, and indicate that the use of intraprostatically injected SPIONs combined with intraoperative use of a handheld magnetometer forms a reliable replacement for the radioactive approach in PCa patients. In a meta-analysis of SLN biopsy in breast cancer using the same magnetic technique for SLN biopsy, pooled data could show that the magnetic technique was non-inferior to the standard technique (*z* = 3.87, *p* < 0.001), too. The mean detection rates for the technetium-based standard and magnetic techniques alone were 96.8% (94.2–99.0%) and 97.1% (94.4–98.0%), respectively. Mean false-negative rates were 10.9% (range 6–22%) for the standard technique and 8.4% (2–22%) for the magnetic technique. Using a random-effects model, the total number of LNs removed was significantly higher with the magnetic technique (*p* = 0.003) [18]. Similarly, in the SentiMag Pro II Study, the number of SLNs removed (median 9, IQR 6–12) was higher than described in our own previous studies using the technetium-based procedure, in which 6 SLNs (median; IQR 4–8) were resected [5]. A possible cause for this could be the smaller size of SPION (60 nm; ^99m^Tc nanocolloid: <80 nm) which could result in marking of secondary landing sites, too [19].

There are various possible causes limiting the effectiveness of intracorporal detection of SPION-marked SLNs using a magnetometer. One problem of intracorporeally detection is that adipose tissue surrounding SLNs can limit the proximity of the probe to the LN, resulting in insufficient exposure of the LN. An insufficient measurement of the magnetic signal can be the result. Moreover, the presence of tissue in the vicinity of the probe tip of the magnetometer and the LN reduces the in vivo magnetic signal because of the negative magnetic susceptibility of surrounding tissue [20]. The limited spatial resolution of the SentiMag^®^ probe (~20 mm) could restrain the differentiation of SLN signals from the background signal from the injection site. In the near future, the higher resolution of novel probes using magnetic tunneling junction techniques (resolution ~4 mm) could lead to a sustainable improvement in intracorporeally SLN identification [20]. In addition, the now available possibility of preoperative localization of magnetically marked SLNs using magnetic resonance imaging (MRI) could further improve intraoperative detectability [10].

Other disadvantages of the magnetometer-guided procedure are the need for frequent balancing of the magnetic baseline level and the requirement for use of plastic or other non-magnetic retractors during surgery [18]. This circumstance also complicates the development of laparoscopic probes for magnetic SLN detection [21]. Another limitation of the magnetic technique is the maximum depth at which the magnetic signal can be detected. Currently available handheld magnetometers do not reach the same depth as a gamma probe (e.g., SentiMag: ~20 mm; TAKUMI^®^ magnetic probe: 10 mm), which can have consequences for the identification of deeper nodes [22,23]. In the MELAMAG Trial, patients with melanoma who underwent SentiMag-guided SLN biopsy in the axillary basin had a lower SLN identification rate than those who required SLN biopsy in the groin basin, seemingly owing to SLNs being located deeper in the axilla compared with the groin [23]. In principle, it must be taken into account that unlike radioactive marking or the use of a gamma probe, contact of the magnetometer with the tissue is mandatory. This circumstance must be considered during intraoperative use of magnetometers and requires a certain adaptation to the radioactive approach.

Preoperative SLN identification offers the surgeon a roadmap with solid information on individual location of draining LNs. So far, lymphoscintigraphy cannot be undertaken without a radioisotope tracer. However, preoperative SLN visualization on MRI after intraprostatic SPION injection was demonstrated recently in PCa patients and could replace the conventional lymphoscintigraphic procedure [10]. MRI is highly sensitive to very small concentrations of SPION and very small SLNs could be visualized. The high spatial resolution of MRI allows the for differentiation of SLNs adjacent to each other, which appear as one hotspot in lymphoscintigraphy. In contrast, the spatial resolution of radioisotope-based lymphoscintigraphy is quite limited (~7–8 mm), which makes identification of smaller LNs, typical of pelvic LNs, difficult. The differentiation of SLNs, especially in the periprostatic, presacral, and perirectal region, is difficult because of high periprostatic activity and excreted radiotracer in the bladder [19].

Another important advantage for routine clinical use of the magnetic sentinel method is that this non-radioactive procedure does not require a radiation-controlled area or imaging facilities and can be safely implemented without facilities’ restrictions in large parts of the world. For example, in Japan, currently, approximately 30% of the facilities do not have a radiation-controlled area [22]. Furthermore, SPION tracer is retained in LNs for a longer time, allowing for delayed sLND (up to 7 days for Sienna+ in breast cancer), which facilitates the patient planning. There is no dependence in radioactive isotope supply, which in recent years has been problematic because of cutbacks in production. Furthermore, the exposition of patients and surgical staff to ionizing radiation is avoided.

In the SentiMag Pro II study, in 22.2% (*n* = 4) of LN-positive cases, metastases were also found in non-SLNs. All four cases were patients with high aggressive PCa (PSA > 40 ng/mL, Gleason score ≥ 8), in accordance with previous reports showing poorer outcomes for sLND with highly aggressive tumors [24]. One fundamental problem of the SLN approach is that fully metastasized LNs or blocked lymph pathways can redirect the tracer, as has already been described for lymphatic spread, and LNs not detected by sLND might already be connected downstream [25]. However, magnetometer-guided sLND may detect LN metastases outside the established eLND template. For example, in the SentiMag Pro II study, 7% of positive nodes were detected in the presacral region. Others demonstrated that 7% of preoperatively SLNs were identified in the presacral region, and 8% of patients with LN metastasis would have been missed if an LND in the presacral region had not been performed [26]. Thus, if the goal is to remove as many positive LNs as possible and not just SLNs, sLND must be combined with eLND in high-risk PCa patients.

The limitations of this study include those inherent to the selection bias associated with surgical series from a single institution and a small sample size. In terms of limitations, it should be noted that the study center that conducted the SentiMag Pro II trial has a very high level of expertise in sLND approaches, which may have introduced bias. However, it remains to be emphasized that the staging reliability and rates of LN metastasis detected by magnetometer-guided sentinel lymphadenectomy in the monitored sample compare well with results from other sLND series [4]. To overcome these limitations, multicenter studies with a larger number of cases should be performed. In addition, a direct comparison of the new magnetic procedure with the radioisotope-guided approach, which can be accomplished by injecting both tracers, as performed by others in breast cancer patients, would be desirable [12,27]. However, our ethics committee did not allow us to perform this in the SentiMag Pro II study.

Initial studies on the magnetic sentinel method have also been carried out on several other tumor entities, which are to be continued in a comparable manner. Recently, the feasibility of intraoperative magnetic SLN identification using the SentiMag system in penile cancer patients could be shown in two pilot studies [14,28]. In addition, our working group was able to demonstrate the visualization of SLNs using MRI after peritumoral SPION injection in penile cancer [14]. Klimczak et al. compared the efficacy and safety of SLN detection in eight vulvar cancer patients using the SentiMag system with the standard radioactive tracer and reported a high detection rate (100%) and complete concordance to the standard procedure [29]. The magnetic sentinel technique is also feasible for SLN biopsy in melanoma with a high SLN identification rate [23]. However, comparing with the standard dual technique, the predefined non-inferiority margin was not reached in the MELAMAG Trial [23]. Furthermore, initial data on the use of magnetometer-guided SLN detection in lung cancer and colorectal cancer were reported [30,31].

In the future, non-invasive techniques could supplement surgical LN staging, including the sentinel procedure, or partially replace LND in longer term. Recent work highlights circulating tumor DNA (ctDNA) present in the blood as a supplemental, or perhaps an alternative, source of DNA to identify the clinically relevant cancer mutational landscape. This noninvasive approach may facilitate repeated monitoring of disease progression and treatment response. Potential applications of this noninvasive information in tumor staging, treatment, and disease prognosis are under discussion [32]. For example, Yang et al. propose the development of a staging system including analysis of ctDNA from liquid biopsy (B) (TNMB staging system) to enhance the current TNM cancer staging system. This model assumes that there is no LN metastasis in the case of undetectable ctDNA mutations in the blood [32]. A systematic metanalysis could demonstrate that cell-free DNA (cfDNA) levels detected by liquid biopsy can be predictive of axillary LN metastasis in patients with breast cancer [33]. Circulating epithelial tumor cells (CTCs) themselves can also provide noninvasive insights in the state of metastasis. However, CTCs are rare, comprising as few as 1–10 cells per 109 hematological cells, and CTC shedding from a solid tumor into the bloodstream is a highly discontinuous process. Thus, the isolation of CTCs with high purity is still a very significant challenge [34]. To meet this challenge, Loeian et al. developed a nanotube-CTC-chip for isolation of tumor-derived epithelial cells (CTCs) from peripheral blood, with high purity, by exploiting the physical mechanisms of preferential adherence of CTCs on a nanotube surface [34]. In clinical studies they used this nanotube-CTC-chip to isolate CTCs with high purity from breast cancer patients. In this initial investigation, CTCs were captured in patients that were LN-positive and negative. However, an apparent increase in CTC counts between early stage (stage 1–3) and advanced disease (stage 4) using the nanotube-CTC-chip could be shown. In order to be able to use the chance, which these new non-invasive techniques offer also for the selection of LN-positive patients, their results should be correlated in further investigations also with the results of targeted sentinel lymphadenectomy showing a high reliability in the detection of patients with LN metastases.

## 4. Materials and Methods

### 4.1. Study Design and Patients

The prospective monocentric SentiMag Pro II study (German Clinical Trials Register: DRKS00007671) investigated the diagnostic accuracy of a novel technique for intraoperative SLN detection in PCa patients, using SPIONs and a handheld magnetometer.

Fifty patients with intermediate- or high-risk PCa (European Association of Urology risk group definitions) scheduled for open radical retropubic prostatectomy and pelvic LND between February and September 2015 were included in this study [35]. Inclusion criteria were a PSA level ≥10.0 ng/mL and/or a Gleason sore ≥7. Exclusion criteria included a known intolerance or hypersensitivity to iron or dextran compounds, iron overload disease, a pacemaker or other implantable device in the chest wall, hormonal treatment, and previous prostate surgery.

### 4.2. Magnetic Superparamagnetic Iron Oxide Nanoparticle (SPION) Tracer

The SPION tracer (Sienna+^®^) used in this study is a component of the SentiMag^®^ system (Endomagnetics Ltd., Cambridge, UK). This system for marking and identifying SLNs comprises a handheld magnetometer, the SentiMag^®^ unit, and the Sienna+^®^ magnetic tracer. All are Conformité Européene (CE) certified as class IIa medical devices. The particles have a carboxydextran coating and a mean hydrodynamic diameter of 60 nm. Sienna+ has comparable functional properties to that of ^99m^Tc nanocolloid, because upon interstitial injection the tracer flows through the lymph system and gets trapped in SLNs in the same manner as the radionuclide.

### 4.3. Tracer Injection

The sentinel technique in PCa differs from that in other tumor types. In breast cancer and malignant melanoma, a well-directed peritumoral injection is used to observe the lymphatic drainage of the tumor only. In PCa, which commonly occurs as a multifocal malignancy, it is not known with absolute certainty from which part of the organ the metastatic spread originated, or which lesion is the index lesion. Therefore, the aim of prostate lymph scintigraphy must be the imaging of all the primary draining LNs of the prostate, which must therefore include the SLN of the cancer.

In this study, one urologist injected 2 mL of SPION (Sienna+) into the prostate of patients using transrectal ultrasound guidance 24 h before surgery. Based on our examinations and those of others, the tracer was evenly spread as three deposits on both sides of the prostate in all cases, as described previously [13].

### 4.4. Magnetometer-Guided sLND, eLND, and Histopathological Examination

Patients underwent magnetometer (SentiMag)-guided sLND and eLND, followed by radical retropubic prostatectomy. All cases were performed by two high-volume surgeons, who applied the same anatomical template during eLND. The eLND template included the area along the external iliac vessels, with the distal limit being the femoral canal. Proximally, eLND was carried out to, and included, the bifurcation of the common iliac artery. All lymphatic fatty tissue along the internal iliac artery and within the obturator fossa and the area dorsal to the obturator nerve was removed, as described by Weingärtner et al. [36]. The lateral limit consisted of the pelvic sidewall, while the medial dissection limit was defined by the perivesical fat.

During sLND, all metal retractors were removed from the surgical field and polymer retractors (SUSI^®^, Aesculap^®^; B. Braun Melsungen AG, Melsungen, Germany) were used to avoid interference with the magnetometer when detecting SLNs with the SentiMag probe. All SLNs detected by the SentiMag were removed, with each magnetically active LN being considered as an SLN. In addition, the magnetic activity of all LNs was measured ex vivo. For surgical reasons, LNs other than SLNs directly adjoining and adhering to SLNs were also removed if in situ separation was not possible. In such cases, LNs were macroscopically detected (tactile and visually) ex vivo and the surgeon separated them from each other or from the containing fibro-fatty tissue. Thereafter, eLND was conducted to remove the remaining lymphatic fatty tissue from the above-named regions. Afterwards, LNs were macroscopically detected and separated from those containing fibro-fatty tissue by the surgeon.

Postoperatively, all LNs were detected and separated by the surgeon (SLNs and non-SLNs), cut into 3 mm transverse sections, and routinely processed and embedded in paraffin, while 4–5 µm-thick sections were further cut and stained with hematoxylin-eosin.

### 4.5. Outcome Measures of Magnetometer-Guided sLND

As established by our and other working groups, and in line with the results of an international sentinel consensus meeting, the diagnostic accuracy of sLND was assessed using conventional eLND in the same cohort as the reference standard [4,37]. Compliance with this standard ensures that our results can be compared with the results of other sentinel techniques.

The outcomes used to analyze the diagnostic test accuracy were detection rate (patients with at least one detected SLN/total number of patients operated on), sensitivity, specificity, PPV, NPV, false-positive, and false-negative rates, with all being measured at the patient level. False-negative cases were defined as patients with a histologically negative SLN, whilst cancer was found in other LNs. False-positive cases were defined as patients with SLNs containing metastases found outside the eLND template, while the eLND template did not reveal any metastases [4]. Thus, the false-positive rate provides a measure of the additional diagnostic value of sLND over and above eLND (false-negative on eLND).

A 2 × 2 table with sLND as the index test and eLND as the reference standard was used to calculate sensitivity, specificity, NPV, and PPV. Additionally, the anatomical distributions of detected LN metastases and identified SLNs were analyzed.

### 4.6. Ethical Approval

All subjects gave their informed consent for inclusion before they participated in the study. The protocol followed in this study was in accordance with the ethical standards of the 1964 Helsinki Declaration and its later amendments. The protocol was approved by the Medical Chamber of Lower Saxony, Germany (Bo/24/2014).

## 5. Conclusions

After interstitial injection, SPIONs are filtered and retained in SLNs allowing intraoperative identification of SLNs using handheld magnetometers. Based on these characteristics, SPIONs have been successfully applied for marking and intraoperative detection of SLNs in breast cancer to overcome the drawbacks of the radioisotope-based sentinel procedure. In several studies including breast cancer patients, the magnetic SentiMag technique for SLN biopsy was non-inferior to the standard method. The results of this prospective clinical trial suggest that the magnetometer-guided radiation-free sentinel procedure could be a reliable replacement for the established radioisotope-based approach in PCa patients who are at intermediate- or high-risk for LN involvement, too. With the aim of detecting all LN metastases in high-risk patients, sLND should be performed in addition to eLND, because of its additional diagnostic value and the detection of LN metastases outside the extended template. The reliability of intraoperative SLN detection using the SentiMag system requires verification in further multicentric studies, including comparisons with other new magnetometer modalities (e.g., probes with permanent magnet and hall sensor or magnetic tunneling junction sensors), which were presented recently. In addition, the promising initial results in other tumor entities such as melanoma, vulvar, and penile cancer should also be pursued.

## Figures and Tables

**Table 1:** Patient characteristics.

Characteristics	Overall	Patients with Negative LNs	Patients with Positive LNs
	*n* = 50	*n* = 32 (64%)	*n* = 18 (36%)
Age, years (median)	69.5	68.5	71.5
IQR	64–73	64–73	64.5–73
Total PSA, ng/mL (median)	10.8	9.8	12.0
IQR	7.4–19.2	6.9–14.7	8.3–30.1
Number of LNs removed (median)	18	19	17.5
IQR	15–23	15–23	16–22
Number of SLNs removed (median)	9	9	10
IQR	6–12	5–11	7–12
Number of positive LNs (median)			2
IQR			1–3
Tumor stage (%)			
T1c	28 (56)	22 (68.8)	6 (33.3)
T2a	2 (4)	1 (3.1)	1 (5.6)
T2b	6 (12)	4 (12.5)	2 (11.1)
T2c	12 (24)	5 (15.6)	7 (38.9)
T3	2 (4)	0 (0)	2 (11.1)
Biopsy Gleason score (%)			
6 (3 + 3)	8 (16)	8 (25.0)	0 (0)
7 (3 + 4)	26 (52)	18 (56.3)	8 (44.4)
7 (4 + 3)	6 (12)	5 (15.6)	1 (5.6)
≥8	10 (20)	1 (3.1)	9 (50.0)
Postoperative Gleason score (%)			
6 (3 + 3)	2 (4)	2 (6.3)	0 (0)
7 (3 + 4)	23 (46)	19 (59.4)	4 (22.2)
7 (4 + 3)	14 (28)	8 (25.0)	6 (33.3)
≥8	11 (22)	3 (9.4)	8 (44.4)
Pathologic stage (%)			
pT2	24 (48)	22 (68.8)	2 (11.1)
pT3a	12 (24)	7 (21.9)	5 (27.8)
pT3b	12 (24)	3 (9.4)	9 (50.0)
pT4	2 (4)	0 (0)	2 (11.1)

IQR, Interquartile range; (S)LN, (sentinel) lymph node; PSA, prostate-specific antigen.
